# Hydration status, body composition, and anxiety status in aeronautical military personnel from Spain: a cross-sectional study

**DOI:** 10.1186/s40779-021-00327-2

**Published:** 2021-06-02

**Authors:** Alejandra Carretero-Krug, Natalia Úbeda, Carlos Velasco, Juan Medina-Font, Trinidad Trujillo Laguna, Gregorio Varela-Moreiras, Ana Montero

**Affiliations:** 1 Departamento Ciencias Farmacéuticas y de la Salud, Facultad de Farmacia, Universidad San Pablo-CEU, CEU Universities, Urbanización Montepríncipe, 28660 Boadilla del Monte, Spain; 2 Centro de Instrucción de Medicina Aeroespacial, Ejército del Aire, 28850 Torrejón de Ardoz, Madrid Spain

**Keywords:** Hydration status, Military aeronautical personnel, Body composition, Anxiety status, Water intake

## Abstract

**Background:**

An adequate hydration status is critical to ensure efficiency during mental and physical activities. Our goal was to assess the hydration status of a Spanish group of aeronautical military men and to determine the association of hydration status with body composition and anxiety.

**Methods:**

A total of 188 men were evaluated through a validated hydration questionnaire, anthropometric and biochemical parameters, and an anxiety questionnaire. Based on these methods, the criteria of hydration were established.

**Results:**

Of the total sample, 81% met the hydration criteria (urine color = well hydrated, water balance ≥ 0 ml, and total water intake/weight ≥ 35 ml/kg), and 19% did not meet the hydration criteria (urine color = not sufficiently hydrated or dehydrated, water balance < 0 ml, and total water intake/weight < 35 ml/kg). Subjects not meeting the hydration criteria had lower urine pH, negative water balance, and lower water intake. The latter also had higher anxiety status (score = 4 vs. 3, *P* = 0.026), weight [(84.7 ± 10.5) vs. (80.5 ± 10.2) kg], body mass index [(26.3 ± 3.1) vs. (25.2 ± 2.8)]kg/m^2^, body fat [(22.3 ± 5.6) vs. (18.3 ± 6.5)] %, urine specific gravity, and urine color. Using a logistic binary regression model, hydration status was related significantly with the percentage of body fat (*P* = 0.004), but no relation was found with age, comorbidities, or medications.

Furthermore, total water intake/weight was positively correlated with percentage of body water (*r* = 0.357, *P* = 0.000) and negatively with body fat (kg) (*r* = − 0.427, *P* = 0.000), percentage of body fat (*r* = − 0.405, *P* = 0.000), and waist/hip ratio (*r* = − 0.223, *P* = 0.002). Based on a linear regression model, total water intake/weight was related significantly with percentage of body fat (*P* = 0.001) and percentage of body water content (*P* = 0.035). No relation was found, however, with waist/hip ratio, age, comorbidities, or medications.

**Conclusions:**

These findings all suggest a relationship between hydration status and body composition but also set the bases for future studies that relate hydration status and anxiety status. These results can be used to improve the hydration status and body composition of military personnel.

## Background

Optimal functioning of the human body requires an adequate hydration level [1] given by a state of water and electrolyte balance [2, 3]. The human body has evolved to include mechanisms to ensure sufficient water content by adjusting intake and waste. Water balance (WB) is determined by intake (water contained in beverages and foods, metabolic water) and waste (urine, stool, skin, expired air from the lungs). Failure of these mechanisms and subsequent impairments in WB may produce severe problems [1, 4]. Mild, acute, and chronic dehydration can have important implications for human behavior and health. Such implications include short-term memory deficits, impaired problem-solving ability, degraded ability to concentrate, reduced cognitive performance, diminished visual-motor tracking, and cardiovascular and kidney diseases [5–8]. Therefore, maintaining an adequate state of hydration is critical to ensure efficiency during mental and physical activities and plays a key role in preventing accidents by minimizing human error [9]. Human error is considered to be the main cause of flight accidents and crashes [10]. In fact, human factors are cited in approximately 70–80% of all accidents in aviation [11]. The complexity of air force missions requires the exercise of a diversity of functions and tasks. The aeronautical military population is vulnerable because of its frequent exposure to a state of hypohydration due to constant physical training; furthermore, military personnel lack the option of choosing their own desired food, as it is provided in military rationing options during base transfers and/or military exercises. In addition, military personnel experience rapid changes in climatic zones with no acclimatization time [12]. There are several guidelines in the scientific literature for establishing recommendations for water requirements in order to maintain an adequate WB [13–16]. However, WB must be individualized for best results [17], because water intake (WI) and water elimination (WE) depend on unique personal characteristics (e.g., age, sex, disease states), environmental conditions, and physical activity levels that vary in different countries or population groups [18, 19]. It has been noted that the percentage of the general population with inadequate WI varies from 5 to 35% among European countries [20]. In Spain, the ANIBES Study (2013) [21] clearly showed that individuals had inadequate levels of total water intake (TWI) when compared with European Food Safety Authority (EFSA) reference values (2.0 L/d for females, and 2.5 L/d for males) [13].

To date, no study has assessed the hydration status (HS) of military personnel in Spain. For these reasons, the purpose of the present study was to evaluate, for the first time, the hydration status of an aeronautical military personnel group in Spain, through a complete and validated questionnaire of hydration [22] as well as anthropometric and biochemical parameters. As a secondary goal, we aimed to investigate the possible relationship between the HS, body composition, and anxiety status, which may affect the work performance and certain abilities in this special population.

## Methods

The present study was a cross-sectional observational study of a sample of Spanish aeronautical military personnel who attend annual medical check-ups at the official *Centro de Instrucción de Medicina Aeroespacial* (CIMA), Madrid. From March 2018 to June 2018 (average temperature of 9.2 °C in March, 12.8 °C in April, 16.6 °C in May, and 21.7 °C in June), subjects from all over Spain were informed of the study and invited to participate. The inclusion criteria were good mental and physical health (determined through various medical consultations included in the regular medical check-up). Exclusion criteria were (a) diseases or any special health conditions that limited their flight license, and/or (b) diseases related to hydration status, including renal impairment, urinary tract infection, water balance disease, and diabetes. This study was conducted according to the guidelines laid down in the Declaration of Helsinki, and all procedures involving human subjects were approved by the Clinical Research Ethics Committee of the CEU San Pablo University (Madrid). The corresponding ethics code is 119/16/08. All the aeronautical personnel who participated in the study did so on a voluntary basis, and their written informed consents were obtained prior to the study.

### Recruitment

The military aeronautical personnel attended the CIMA for a 2- to 3-day medical check-up period. Recruitment started the first day of medical check-ups for each group and included a volunteer recruitment briefing with infographics of the procedure of the study. From March 2018 to June 2018, volunteers that expressed interest in participating in the study received the study protocol in writing as well as verbal responses to any questions. Of the total number of subjects, 6% did not consent to participate in the study or could not participate because they did not meet the inclusion criteria.

### Study protocol

During the first day, the participants completed the self-reported questionnaires under the supervision of trained dietitian personnel.

The “hydration status questionnaire” (HSQ), validated by the Nutrition and Food Science research group of CEU San Pablo University [22] included the following: a) personal information; b) medical history; c) hydration habits and knowledge; d) a water, beverages, and food frequency questionnaire (WBFFQ); and e) water elimination (WE) information. To estimate water output, three elimination pathways were taken into account: skin, kidneys, and the digestive system. Urination and defecation were recorded on the basis of frequency, and to calculate sweating, a 10-point scale was used for both physical activity and sedentary conditions [23]. This information allowed for the assessment of the estimation of WB.

A specifically designed questionnaire evaluating physical activity through a brief register of working day and non-working day activities was used. In this questionnaire, a total of 24 h were divided into sleeping, eating, work activity, sitting or lying (reading, watching TV, etc.), walking or other modes of transport, other activities (specifying domestic tasks, playing with kids, etc.), and sports (specifying which). The total daily energy expenditure (TDEE) was calculated according to their respective metabolic equivalent (MET) intensity levels [24]. This TDEE enabled the estimation of the sweat produced by the physical activity in order to estimate WE.

An anxiety questionnaire (State Trait Anxiety Inventory (STAI)) [25] was used, where a) STAI—State assesses a transitory emotional state, and b) STAI—Trait indicates an anxious propensity. The score for each scale can range from 0 to 10, where upper scores indicate higher levels of anxiety.

Furthermore, volunteers provided an afternoon (14:00–16:00) p.m. spot urine sample, which has an equivalence with the 24 h value of urine [the mean differences (95% confidence interval) between 24 h and spot urine were 0.0014 (− 0.0028 to − 0.0001)] [26], in which urine pH and urine specific gravity (USG) were determined through the use of a Spinreact™ urine stick test, and urine color via the Urine Color Chart [27]. Results of USG were compared with reference values of hydration biomarkers in urine afternoon spot samples [4] [euhydration values: specific gravity = (1018–1020) g/L], and urine color were compared with reference values of the Urine Color Chart [27]. As indicated by the urine color chart, values 1, 2, and 3 correspond to well hydrated (WH), 4, 5, and 6 correspond to not sufficiently hydrated (NSH), and 7 and 8 correspond to dehydrated (DH).

Armstrong et al. [28] indicate that to estimate the hydration status information, two or more hydration assessment techniques are needed. Therefore, urine color, WB, and total water intake/weight were used to classify study participants with respect to hydration adequacy. Participants that met the following parameters were identified as meeting the hydration criteria: urine color = WH [27], WB ≥ 0 ml, and total water intake/weight ≥ 35 ml/kg weight [29]. Participants that met urine color = NSH or DH, WB < 0 ml, and total water intake/weight < 35 ml/kg weight, were classified as not meeting the hydration criteria. Note that the hydration criteria in this study represented benchmarks for grouping individuals and were not treated as criteria for dehydration or any clinical diagnosis.

During the second day of the study, the subjects completed the remaining tests under fasting conditions of water and food. First, blood samples were taken for hemoglobin, hematocrit, and erythrocyte analysis. All the determinations were performed in a Cobas Core 501 autoanalyzer (Roche-Hitachi, Switzerland). Then, anthropometric parameters were determined according to the protocol of the International Society for Advanced Kinanthropometry (ISAK) [30]. Specifically, measurements of weight, height, waist, and hip circumferences were taken. The waist/hip ratio was calculated from the equation waist (cm)/hip (cm), following the World Health Organization (WHO) criteria [31]. Body mass index (BMI) was calculated from the equation weight (kg)/height^2^ (m^2^), and compared to WHO reference values [32]. Body fat percentage and body water percentage were determined by Bioelectrical Impedance (BIA) (InBody 770®, Spain). Excessive body fat was defined according to body fat ranges for standard adults, as reported by Gallager et al. [33], whereas body water percentage was compared with standard ranges between 55 and 65% [34].

### Statistical analysis

Given that the size of target population (personnel from the Spanish Air Force) is confidential and not publicly available, it was impossible to calculate the exact sample size required for representativeness.

Normality of variables was tested using the Kolmogorov–Smirnov test. In light of the results obtained, descriptive values were presented as mean ± standard deviation (SD) or median [inter-quartile range (IQR)]. Differences between normally distributed variables were derived using Student’s *t*-test. For non-parametric measurements, Mann–Whitney *U* tests were applied. Urine color was tested using Chi-square test. Differences were considered significant at *P* < 0.05.

Correlation analysis was performed using Spearman’s coefficient or Pearson’s coefficient according to variable normality. All the statistical analyses were performed using SPSS 24.0 Software (IBM Corp., Armonk, NY, USA).

A linear regression model was constructed to explore the associations between water intake adjusted by body weight as a dependent variable, and the body composition variables age, co-morbidity, and medications as independent predictors. Furthermore, a logistic binary regression model was used to explore associations between hydration status and body composition variables (body fat percentage), age, co-morbidity, and medication as independent predictors.

## Results

### Anthropometric measurements, blood, and urine markers

A total of 188 healthy males aged 23 to 56 years ultimately participated in the study [67.6%, (20–39) years, and 32.6%, (40–59) years], with a TDEE of 3158.7 (333.9) kcal/d. Of the total sample, 18% presented co-morbidities, and 16% took medications unrelated to hydration status. The body weight of the participants was (81.6 ± 10.3) kg, while body height was (178.7 ± 6.2) cm. A total of 50.0% of the sample showed normal weight values, 42.6% were overweight, and 7.4% were categorized as obese according to their BMI values. Mean BMI (25.4 ± 2.9) kg/m^2^ was considered in the lower range of overweight according to reference values. However, we observed a percentage of body fat (19.1 ± 6.5) % established as normal in adult men. Furthermore, the waist/hip ratio (0.87 ± 0.05) in the total sample showed no risk factors for cardiovascular disease. Percentage of body water (60.4 ± 7.5)% in the total sample was also in the range of normal values 55–65% [34].

Moreover, blood indices [erythrocytes, (5.2 ± 0.4) million/μl; hemoglobin, (15.2 ± 1.1) g/dl; and hematocrit, (44.7 ± 2.5)%] were within the physiological range [35], as were the urine markers [36] [pH, 6 (5, 7); and specific gravity, 1020 (1015, 1020) g/L].

### Water intake, water elimination, and water balance

Water from beverages (2709.5 ± 943.6) ml/d, water from foods (795.5 ± 413.7) ml/d, total water intake (3508.9 ± 1112.1) ml/d, water elimination (3402.4 ± 1086.5) ml/d, water balance (88.5 ± 1294.3) ml/d, and total water intake/weight (43.7 ± 14.9) ml/kg were estimated using the HSQ. Total water intake/weight was correlated positively with percentage of body water (*r* = 0.357, *P* = 0.000) and negatively with body fat (kg, *r* = − 0.427, *P* = 0.000), percentage of body fat (*r* = − 0.405, *P* = 0.000), waist (*r* = 0.390, *P* = 0.000), hip (*r* = − 0.401, *P* = 0.000), and waist/hip ratio (*r* = − 0.223, *P* = 0.002).

To evaluate the independent relation between total water intake/weight and body composition variables (waist/hip ratio, percentage of body water, and percentage of body fat), age, co-morbidities, and medications, a linear regression model was used (Table 1). The total water intake/weight was significantly related with percentage of body fat (*P* = 0.001) and percentage of body water (*P* = 0.035). No relation was found with waist/hip ratio, age, comorbidities, or medications.

**Table 1:** Water intake adjusted by body weight in a linear regression analysis

Item	B	SE	*t*	95%CI	*P* value
Waist/hip ratio	−2.330	25.785	−0.090	−53.217, 48.557	0.928
Body water (%)	0.378	0.178	2.122	0.0264, 0.729	0.035
Body fat (%)	−0.706	0.212	−3.334	−1.123, − 0.288	0.001
Age	0.187	0.171	1.091	−0.151, 0.525	0.277
Comorbidity	3.354	5.364	0.625	−7.232, 13.941	0.533
Medication	−0.289	4.981	−0.058	−10.118, 9.541	0.954

*SE* Standard error of B, *95% CI* 95% confidence interval, *r* = 0.198, *R*^2^ = 0.184, and *r* adjusted = 0.166

### Hydration status

It was found that 81% of the total population met the hydration criteria, whereas 19% did not. Table 2 shows TDEE and anthropometric characteristics according to the hydration status. Even though there were no statistical differences in anthropometric data according to HS, subjects that did not meet the hydration criteria had higher weight [(84.7 ± 10.5) vs. (80.8 ± 10.2)] kg, BMI [(26.3 ± 3.1) vs. (25.2 ± 2.8)] kg/m^2^, body fat [(22.3 ± 5.6) vs. (18.3 ± 6.5)]%, and waist/hip ratio [(0.9 ± 0.1) vs. (0.87 ± 0.1)], and lower body water [(58.1 ± 7.2) vs. (60.9 ± 7.5)]% than subjects that met the hydration criteria.

**Table 2:** Total daily energy expenditure and anthropometric characteristics categorized by hydration status ($\overline{x}$ ± *s*)

Item	Not meet hydration criteria (*n* = 36)	Meet hydration criteria (*n* = 152)	*P* value
TDEE (kcal/d)	3196.9 ± 352.4	3149.6 ± 329.9	0.938
Weight (kg)	84.7 ± 10.5	80.8 ± 10.2	0.543
BMI (kg/m^2^)	26.3 ± 3.1	25.2 ± 2.8	0.244
Body water (%)	58.1 ± 7.2	60.9 ± 7.5	0.868
Body fat (%)	22.3 ± 5.6	18.3 ± 6.5	0.551
Waist/hip ratio	0.9 ± 0.1	0.87 ± 0.1	0.171

*P* value derived through Student’s *t*-test

*BMI* Body mass index, *TDEE* Total daily energy expenditure

Results of WI, WE, and WB are presented in Table 3. Subjects that did not meet the hydration criteria had significantly lower water intake from beverages (*P* = 0.002), water from food (*P* = 0.005), total water intake (*P* = 0.000), water balance (*P* = 0.034), and total water intake/weight (*P* = 0.000); than subjects that met the hydration criteria. Also differences were showed in urine markers, between subjects that did not meet the hydration criteria and subjects that meet the hydration criteria: pH [5 (5, 6.5) vs. 6 (5, 7), *P* = 0.023]; specific gravity [1020 (1015,1020) vs. 1020 (1015,1020), *P* = 0.043], and urine color [2 (1, 3) vs. 2 (1, 3), *P* = 0.000]. However, differences in blood indices were not observed in either group [erythrocytes, (5.2 ± 0.3) million/μl vs. (5.2 ± 0.4) million/μl, *P* = 0.953; hemoglobin, (15.3 ± 0.8) g/dl vs. (15.2 ± 1.1) g/dl, *P* = 0.840; and hematocrit, (44.8 ± 2.3)% vs. (44.7 ± 2.2)%, *P* = 0.710].

**Table 3:** Total water intake, water loss, water balance, and total water intake/weight by hydration status ($\overline{x}$ ± *s*)

Item	Not meet hydration criteria (*n* = 36)	Meet hydration criteria (*n* = 152)	*P* value
Water from beverages (ml/d)	1704.4 ± 489.9	2947.6 ± 865.5	0.002*
Water from food (ml/d)	574.1 ± 250.4	847.9 ± 427.7	0.005*
Total water intake (ml/d)	2278.4 ± 473.6	3800.4 ± 1016.4	0.000*
Water loss (ml/d)	3383.3 ± 1130.9	3406.9 ± 1079.5	0.975
Water balance (ml/d)	− 1104.8 ± 996.6	371.1 ± 1193.5	0.034*
Total water intake/weight (ml/kg)	27.1 ± 5.4	47.6 ± 13.8	0.000*
Water loss/weight (ml/kg)	40.1 ± 12.4	42.9 ± 14.9	0.172

*P* value derived through Student’s *t*-test

*Significant differences between groups (Student’s *t*-test, *P* ≤ 0.05)

To evaluate the independent relation between HS and body composition variables (percentage of body fat), age, comorbidities, and medications, a logistic binary regression model was used (Table 4). The HS was significantly related with percentage of body fat (*P* = 0.004), whereas no relation was found with age, comorbidity, or medications.

**Table 4:** Hydration status in a logistic binary regression analysis

Variable	B	SE	β	95% CI	***P*** value
Age	0.001	0.030	1.001	0.943–1.062	0.974
Medication	− 0.250	0.210	0.779	0.267–2.268	0.647
Comorbidity	−0.639	1.586	0.528	0.195–1.426	0.208
Body fat (%)	−0.091	8.130	0.913	0.858–0.972	0.004
Constant	3.431	1.138	30.895	–	–

*SE* Standard error of B, *95% CI* 95% confidence interval, *r* Cox and Snell = 0.72, and *r* Nagelkerke = 0.116

### Anxiety status

Anxiety results showed a low level of anxiety in the total population: score 3 (IQR 2, 5) for STAI-State, and score 2 (IQR 2, 3) for STAI-Trait score. No correlation between STAI (STAI-State and STAI-Trait) and any HS variable was found.

In the HS category, significant differences were noted in the anxiety test STAI-State (*P* = 0.026), where subjects that did not meet hydration criteria had higher scores [4 (3, 5) vs. 3 (2, 4)] than the group that met the hydration criteria. However, no differences were shown in the anxiety test STAI-Trait between subjects that did not meet hydration criteria and subjects that met the hydration criteria [2 (2, 3) vs. 2 (2, 3), *P* = 0.312].

## Discussion

The present study represents a unique exploration of HS on aeronautical military personnel in Spain, as well as the association of this variable with body composition and anxiety status.

The main findings of our study indicate that TWI was adequate for the majority of the population, in accordance with recommendations established by WHO (3.7 L/d) [16], the National Health and Medical Research Council (3.4 L/d for males) [14], and the Institute of Medicine of the United States of America (IOM), which suggests 3.7 L/d for males [37]. Nevertheless, they consumed approximately 40% more TWI than the EFSA WI reference values for men (2.5 L/d) [13] and the Spanish Society of Community Nutrition (SENC) recommendations [38]. Many studies take into account only the water from beverages but not water intake from all sources (beverages plus food water intake) [39, 40]. Previous studies of the general European population found TWI in the range of 2310 ml/d in Ireland to 3254 ml/d in Greece [41–45]; findings in the Spanish population (ANIBES Study) showed TWI of 1625 ml/d [21], which were much lower than our results. Worldwide, TWI ranged from 2230 ml/d in Japan to 3563 ml/d in the USA [46, 47]. Deviation in water intake may reflect country differences in dietary habits, lifestyle choices, and environmental conditions, but also differences in the selection of methods used to evaluate total water intake [42].

On the other hand, mean daily WE was 3402.4 ml/d for the whole sample. WE data were in accordance with the current literature, which is (1500–3100) ml/d for adults [13, 15]. In addition, the WB was positive for the total sample. WI is usually expressed in absolute units (L/d), even though a given intake of water can be expected to have different effects on the hydration status of small versus large individuals [48]. As such, recent NHANES studies suggest that not accounting for body weight to express WI (ml/kg of weight) may introduce confounding factors and/or cause different results in hydration studies [48–50]. In addition, the Spanish Guidelines of Hydration suggest that the standard requirements of WI are (30–35) ml/kg of weight [29], values that were achieved in the present studied sample participants.

Laboratory data for urine (USG, urine color, osmolality) are considered good markers to evaluate HS [51, 52]. In the present study, urine was measured via color chart in an afternoon urine spot, since USG findings of Bottin et al. [26] suggest that an afternoon sample may be an accurate and practical tool for hydration monitoring.

Blood parameters (hematocrit and hemoglobin) were analyzed due to their potential as HS markers [51]; however, no correlation was observed with any other HS variables in the present study. Francesconi et al. [53] investigated military personnel over field training and reported that even when the subjects had lost more than 3% of their body mass and had a high urine specific gravity, there was no change in hematocrit or serum osmolality measurements. They concluded that plasma volume is regulated by the body in an attempt to maintain cardiovascular stability, and therefore plasma variables are not affected by mild hypohydration until a certain degree of body water loss (at least 3% of body mass loss). Similar findings were reported by Armstrong et al. [54]. In this study urine and blood indices were within the physiological ranges in both groups [35, 36].

In our population, 81% of the participants met the established hydration criteria, and 19% did not meet the hydration criteria. Lower results were observed in Belgian military personnel, 13.6% of the sample were classified as dehydrated (using USG of first urine of the morning) [55]. A US military study showed that 31% of the total sample met the criteria for hypohydration (using USG of first urine of the morning) [12]. Malisova et al. [41] concluded that on average over a seven-day period (using cutoffs of 24 h urine osmolality) in an adult free-living population, 60% were euhydrated, 20% hyperhydrated, and another 20% dehydrated. Furthermore, they observed that euhydrated subjects had a higher total water intake and water intake from beverages but a lower specific gravity and lighter color. These results are mostly in agreement with our study.

Regarding anthropometric parameters, BMI suggested overweight values in the total sample. Conversely, percent body fat was considered in the range of healthy values. Several other studies found similar results and an inability to distinguish the different contributions of body weight and fat and non-fat tissue, which explains why the BMI might overestimate adiposity in muscular and lean bodies in active people [56, 57]. Body water content is an accepted indicator of HS [51]. In this study, this parameter was assessed by BIA and showed normal values [34]. Although no significant differences were observed according to HS, subjects that did not meet the hydration criteria had higher weight, BMI, body fat percentage, and waist/hip ratios than subjects that met the hydration criteria. In addition, total water intake per kg of weight was positively correlated with percentage of body water and negatively with body fat (kg), and percentage of body fat and waist/hip ratio for the total sample, demonstrating a relation between hydration status and body composition; these data were also found in a recent study [58]. Likewise, linear and logistic binary regression analysis showed that percentage of body fat and percentage of body water were independent predictors of water intake normalized by body weight in the study population, and furthermore the percentage of body fat was an independent predictor of hydration status. These findings suggest that an adequate water intake could improve body composition. Nevertheless, age, co-morbidities, and medication were not found to be significant predictors of water intake normalized by body weight and of HS in the linear and logistic regression analysis. Thus, in addition, body composition parameters should be taken into account in hydration status monitoring.

In our study, subjects that did not meet the hydration criteria had significantly higher scores in STAI-State than subjects that met the hydration criteria, indicating a possible relationship between these two variables that should be carefully further evaluated. However, in other studies, the relationship between hypohydration status and degraded mood was consistent [8, 59]. In additional studies, measurements of self-reported changes in mental state have consistently found associations between dehydration and mood in conjunction with changes in performance [60, 61]. Neave et al. [62] tested young adults using a range of cognitive tasks, including attention and working memory, and showed that mood ratings significantly changed when individuals were given water. Individuals reported feeling more “calm” and “alert” immediately after water consumption. These results are in line with those of other young adult studies that found similar reports of “alertness” after water consumption [63].

The strengths of this study include the novelty of researching a vulnerable and high-risk group, such as aeronautical military personnel, as well as the careful protocol and methodology used.

Even though the present study’s protocol was well controlled, some limitations should be acknowledged. Regarding the HS, the selection of hydration markers may have limitations. The most important limitation refers to the impossibility of directly assessing water consumption, since it was estimated through a validated questionnaire. Other limitations include the measurement of USG through reagent strips instead of refractometry, and the non-availability of 24 h urine samples.

## Conclusion

Our results indicate that there is a clear association between hydration status and body composition, but also set the bases for future studies that relate hydration status and anxiety status in the military personnel evaluated. These results can be used to highlight the importance of an adequate hydration status and body composition for military personnel, to maintain physical and mental performance for their professional career and development.

## Data Availability

Not applicable.

## Abbreviations

BIA: Bioelectrical impedance
BMI: Body mass index
CIMA: Centro de Instrucción de Medicina Aeroespacial
DH: Dehydrated
EFSA: European Food Safety Authority
HS: Hydration status
IOM: Institute of Medicine of the United States of America
ISAK: International Society for Advanced Kinanthropometry
IQR: Inter-quartile range
MET: Metabolic equivalent
NSH: Not sufficiently hydrated
SENC: Spanish Society of Community Nutrition
SD: Standard deviation
STAI: State-Trait Anxiety Inventory
HSQ: Hydration status questionnaire
TDEE: Total daily energy expenditure
TWI: Total water intake
USG: Urine specific gravity
WB: Water balance
WBFFQ: Water, beverages, and food frequency questionnaire
WE: Water elimination
WI: Water intake
WH: Well hydrated
